# A Case of Renal Vein Thrombosis Associated With COVID-19 Treated With Rivaroxaban

**DOI:** 10.7759/cureus.29491

**Published:** 2022-09-23

**Authors:** Louise Asleson, Mohammed Zalabani, Mohammad Selim

**Affiliations:** 1 Internal Medicine, Creighton University School of Medicine, Omaha, USA

**Keywords:** rvt, covid-19 associated coagulopathy, covid-19, direct oral anticoagulants (doac), renal vein thrombosis

## Abstract

Renal vein thrombosis (RVT) is a rare form of deep venous thrombosis. It usually involves one or both renal veins and one of their branches. Most cases were reported in patients with nephrotic syndrome or inherited hypercoagulability syndromes. RVT can present with flank pain, hematuria, and acute kidney injury but can also present asymptomatically and be incidentally discovered on abdominal or renal imaging. The management of RVT is usually with warfarin for at least six to 12 months and periodically is continued if the patient is in the nephrotic range. Direct-acting oral anticoagulants (DOACs) have not been well studied in cases of RVT, especially in patients with coronavirus disease 2019 (COVID-19). We present a case of RVT in the setting of COVID-19 that was treated successfully with a DOAC, rivaroxaban, with complete resolution of the thrombus.

## Introduction

The coronavirus disease 2019 (COVID-19), caused by the highly contagious severe acute respiratory syndrome coronavirus 2 (SARS-CoV-2), is most commonly associated with respiratory symptoms, including viral pneumonia. However, the virus has been associated with many unique pathologies, including coagulopathy, with the occurrence of venous thromboembolism being a common finding in patients acutely infected, especially those requiring hospitalization [1]. This includes cases of renal vein thrombosis (RVT), a generally rare diagnosis and most often seen in the setting of nephrotic syndrome. The standard treatment for RVT is warfarin [2]. In this report, we discuss a case of renal vein thrombosis in a patient with COVID-19 whose thrombus was treated with a direct-acting oral anticoagulant (DOAC), rivaroxaban, for three months.

## Case presentation

**Figure 1:**
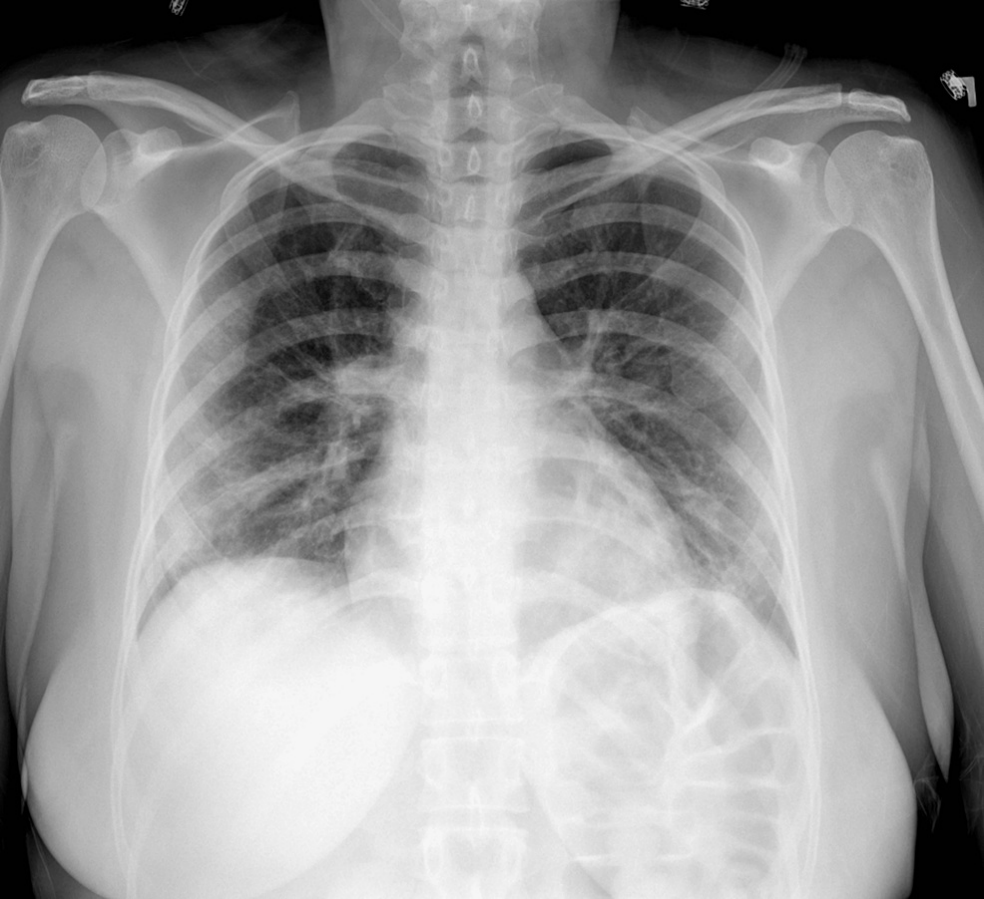
Chest x-ray of a 44-year-old female with COVID-19 viral pneumonia

A 44-year-old female with a past medical history of hypothyroidism and chronic anemia presented to the hospital with shortness of breath and cough. She was hypoxic with an oxygen saturation of 87% on room air. A respiratory pathogen screen was obtained, and the patient was placed on four liters of supplemental oxygen to increase her oxygen saturation to 90-92%. The patient was positive for COVID-19, and a chest x-ray showed bilateral infiltration consistent with COVID-19 viral pneumonia (Figure 1). Laboratory studies were remarkable for anemia and elevated C-reactive protein of 31.8 mg/L. D dimer was not obtained.

**Figure 2:**
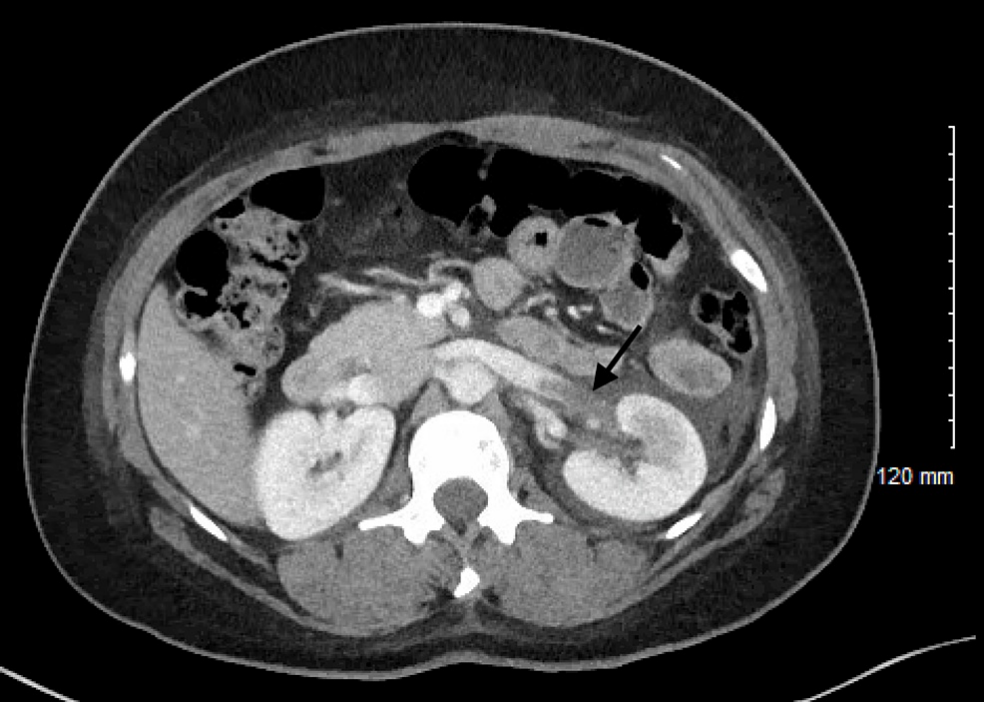
Computed tomography (CT) abdomen and pelvis with contrast showing thrombosis of the left renal vein (arrow)

**Figure 3:**
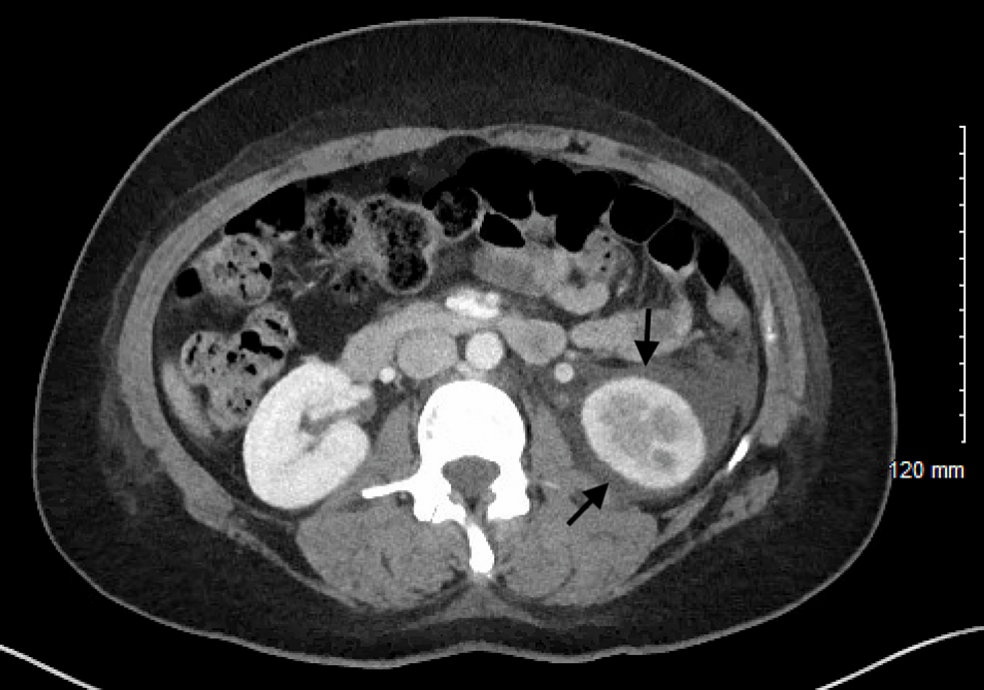
Computed tomography (CT) abdomen and pelvis with contrast showing abnormal hypo-enhancement of the inferior half of the left kidney with prominent perinephric fluid (arrows) in a patient with left renal vein thrombosis

The patient was admitted and started on dexamethasone and remdesivir, completing five days of both medications. The patient was not placed on any prophylactic anticoagulation. Two days into hospital admission, the patient complained of left flank pain. Urinalysis did not show blood or evidence of infection. Kidney function was within normal limits. Computed tomography (CT) of the abdomen and pelvis with contrast showed left renal vein thrombosis with associated abnormal hypo-enhancement of the inferior half of the left kidney with perinephric fluid (Figures 2, 3). The patient was started on therapeutic enoxaparin sodium at one mg/kg twice a day and discharged on oral anticoagulation with rivaroxaban 15 mg twice daily for 21 days, followed by 20 mg daily for three months. Given a lack of other risk factors, the patient’s left renal vein thrombus was considered provoked by her COVID-19 disease. The patient followed up in a clinic three months later, and a repeat CT of the abdomen and pelvis with contrast showed complete resolution of the left renal vein thrombosis. 

## Discussion

Renal vein thrombosis is a rare medical condition that most commonly occurs in the setting of nephrotic syndrome. However, cases are also reported due to inherited hypercoagulability, malignancy, trauma, and infection [2]. The left renal vein is more commonly affected than the right, but two-thirds of patients have bilateral renal vein involvement. Additionally, men have a higher incidence than women. Recently, some cases of RVT have been associated with COVID-19 [3,4]. The association between COVID-19 and thromboembolic complications has been well established and has been attributed to a hypercoagulable state secondary to direct endothelial damage and excessive inflammation caused by the virus [1].

The most common presenting symptoms of renal vein thrombosis are flank pain, hematuria, and nausea/vomiting [2,5]. The presentation is often nonspecific, and suspicion should be high in patients at risk of thrombotic disease. To evaluate for RVT, imaging is necessary for both screening and diagnosis as there are no diagnostic laboratory tests for this condition. Doppler ultrasound is widely used to screen high-risk patients, especially those with nephrotic syndrome or following kidney transplants. However, CT angiography has significantly higher sensitivity and specificity than ultrasound and is the primary modality for diagnosing renal vein thrombosis.

Treatment is generally indicated for acute and chronic RVT. However, some patients with unilateral asymptomatic RVT in nephrotic syndrome can be closely monitored without immediate intervention. Previously, a surgical approach was preferred for RVT, but medical management with anticoagulation has become the mainstay of treatment [2]. The most commonly used agent is warfarin, with a target international normalized ratio (INR) of 2.5 (range from 2.0 to 3.0). Warfarin is well studied in these cases, especially in patients with nephrotic syndrome, and has therefore remained the standard treatment. On the other hand, DOACs have not been well studied in RVT because the phase III studies of these medications have excluded patients with venous thrombosis in atypical locations [2,6]. Thus, the recommendation for thrombi in atypical areas, like the renal veins, is still unfractionated or low molecular weight heparin (LMWH), followed by warfarin. 

However, some small nonrandomized studies have looked at this topic, for instance, in a retrospective study conducted at Cleveland Clinic on eight patients. Six were started on rivaroxaban, and two were started on apixaban. Thirty-six weeks later, follow-up imaging showed partial or complete resolution in 87% of the patients [7]. Another study was published in 2018 that compared rivaroxaban and LMWH in patients with nephrotic syndrome. The study compared two arms of eight patients, with the primary endpoint being the resolution of >90% of the venous thrombosis burden in four weeks. Interestingly, the study found slight superiority and better results in the rivaroxaban arm [8]. Both of these studies support the efficacy of DOACs in treating renal vein thrombosis.

In our case, the patient presented with left RVT in the setting of COVID-19. The patient was started on rivaroxaban with complete thrombus resolution within three months, confirmed with CT imaging. This provides an additional example of successful treatment of renal vein thrombosis with a DOAC. There are currently no studies on using DOACs in treating RVT in COVID-19, and this example will add to the pool of cases. Hopefully, this will encourage the consideration of DOACs in such patients with similar conditions.

## Conclusions

Direct-acting oral anticoagulants have not been well studied in cases of renal vein thrombosis, although there are some small encouraging studies. Furthermore, no studies were done on using DOACs such as rivaroxaban in cases of RVT in patients with COVID-19. Our case here provides an example of safety and success in using rivaroxaban in such cases. However, we believe that more extensive studies are essential to establish the use of DOACs in renal vein thrombosis cases, specifically in patients with COVID-19.
